# Physical Performance and Falling Risk Are Associated with Five-Year Mortality in Older Adults: An Observational Cohort Study

**DOI:** 10.3390/medicina59050964

**Published:** 2023-05-17

**Authors:** Francesco Salis, Antonella Mandas

**Affiliations:** 1 Department of Medical Sciences, and Public Health, University of Cagliari, SS 554 Bivio Sestu, 09124 Cagliari, Italy; 2 University Hospital “Azienda Ospedaliero-Universitaria” of Cagliari, 09042 Monserrato, Italy

**Keywords:** comprehensive geriatric assessment (CGA), physical performance, falling risk, mortality, elderly

## Abstract

*Background*: Falls in older people have a significant impact on public health. The scientific literature has provided evidence about the necessity for older adults to be physically active, since it reduces the incidence of falls, several diseases, and deaths, and can even slow down some effects of aging. The primary aim of our study is to identify if physical performances and risk of falling are related to 1-, 2-, 3-, 4-, and 5-year mortality. Its secondary aim is to establish if people with both severely impaired physical performance and a high risk of falling also present impairment in other geriatric domains. *Methods*: In this prospective study, we enrolled subjects aged 65 years or more, subjected them to comprehensive assessment (including assessment of risk of falling, physical capacities, comorbidities, autonomies in daily living, cognitive abilities, mood, and nutritional status), and followed them for 5 years. *Results*: We included 384 subjects, 280 of whom were women (72.7%), with a median age of 81 years. Our results showed that physical performances and risk of falling are highly correlated to each other (rho = 0.828). After divided the sample into three groups (people without augmented risk of falling and able to perform adequate physical activity; people with moderate risk of falling and/or disability; people with severe risk of falling and/or disability), we found that the more severe the disability and risk of falling were, the more compromised the other geriatric domains were. Moreover, the survival probability progressively increased following the same trend, amounting to only 41% in severely compromised people, 51.1% in moderately compromised people, and 62.8% in people without physical compromise nor an augmented falling risk (*p* = 0.0124). *Conclusions*: Poor physical performance combined with a high risk of falling, correlated with each other, are associated with higher mortality and impairment in multiple domains in older adults.

## 1. Background

The aging of the population represents an increasing concern worldwide [1]. In Italy, life expectancy at birth is 82.6 years, and in 2022 more than 22,000 people have lived 100 or more years [2]. Indeed, it is not only significant from merely a demographic point of view, but also from a public health one. People, who will exponentially increase in number during the next decades [3], may experience changes in muscle mass, balance, and, consequently, risk of injury and disease as they age [4]. It goes without saying that, together with the physical concerns, there are mental and psychosocial issues to be considered, as well as internal medicine issues, since old-aged people are also more likely to experience cardiovascular, neurological, endocrinological, and gastroenterological (among others) diseases [5,6,7]. Additionally, aging also has a significant impact on society in terms of economic costs [8]. As people grow older, they are more likely to require financial assistance, with an increase in demand for services such as long-term care, transportation, and other forms of medical and pharmacological assistance. In this connection, many studies have shown that pharmacological therapy has to deal not only with differences in terms of metabolism in older subjects [9], but also with frequent inappropriate prescriptions [10,11,12], which in turn bring about the increase in the already mentioned risk of falling [13]. As such, comprehensive geriatric assessment has represented and still represents the most useful specialist tool to holistically frame the state of older adults [14,15,16], including the assessment of physical performance [17] and risk of falling [18]. It is known that one of the most significant problems in public health is represented by falls [19], and consequently, bone fractures, which in turn bring hospitalizations, disabilities, and deaths [20,21,22], but it is less known how to identify all the risk factors [23], thus defining what exactly “fall risk” means. It seems to include obvious physical aspects, such as balance and gait, but also cognitive impairment, the assumption of several classes of medications, and environmental matters [24], and can be summarized as defining it as a multifactorial condition which results in negative outcomes, and on the factors for which it is possible to work on in order to avoid them [25]. In such context, even the objective measurement of the sole physical abilities could not be enough to understand and prevent the risk. With the increasing of the global average age, several studies are focusing on the assessment of physical activities [26,27], as physical exercise has been progressively established as a full-fledged therapy [28,29,30]. Physical training proved to be effective even in frail older people in reducing the risk of developing musculoskeletal diseases, muscular weakness, and preventing falls [31]. Distancing from mere osteoarticular matters, physical exercise also reduces metabolic diseases [32], cardiovascular accidents [33], and cognitive impairment [34], not to mention quality of life [35] and depressive symptoms [36]. Unfortunately, it has been reported that, nowadays, some people still believe that training leads to poor benefits, and, on the contrary, can even be dangerous [37]. Current scientific evidence suggests that adequate exercise, in terms of quantity and quality, can even partially reverse some negative effects of aging [38]. Unfortunately, the global socioeconomic disparity is also reflected in global activity inequality, which represents a predictor of obesity prevalence [39].

Since poor physical capacities and the co-presence of several risk factors for falling are indeed multifactorial [25,40], apart from therapeutic interventions, it is necessary to early identify people at greater risk and understand the long-time outcomes the experience.

Taking the above into consideration, we decided to design the present prospective study, the primary aim of which is to identify if physical performance and risk of falling is related to 1-, 2-, 3-, 4-, and 5-year mortality, and the secondary aim of which is to establish if people with both severely impaired physical performance and a high risk of falling also present impairment in other geriatric domains.

## 2. Methods

### 2.1. Design of the Study

This prospective observational cohort study included subjects who were evaluated at the Geriatric Outpatient Service of the University Hospital of Monserrato, Cagliari, Italy, from January 2010 to December 2017, and followed for sixty months.

### 2.2. Study Size

The given confidence level: 95%, confidence interval: 5%, standard deviation (SD): 0.5, Z-score (z): 1.96, and error margin (e): 5%, according to the following formula:

$$N=\frac{z^{2}*SD\;\left(1-SD\right)}{e^{2}}$$

The final sample (N) consisted of 384 subjects.

### 2.3. Inclusion Criteria

Subject age ≥ 65 years; having been subjected to two tests assessing physical performance: Physical Performance Test (PPT) and Performance Oriented Mobility Assessment (POMA)

### 2.4. Exclusion Criteria

Comorbidity Index Rating Scale (CIRS) > 30.

### 2.5. Assessment

The enrolled subjects were evaluated with the following assessments:

- PPT [17], for the assessment of physical performance status. Scores ≥ 20 are indicative of adequate physical capacities, 11–19 indicate moderate disability, and <11 indicate severe disability. It is divided into items, each of which explores the ability to perform a standardized activity, namely, writing a sentence, simulating eating, putting a book on a shelf, putting on and removing a jacket, picking up a coin from the floor, turning 360°, and walking for 15 m: 0 to 4 points are given to each item according to the time spent or the confidence in performing the task.
- POMA [18], for the assessment of the risk of falling. Scores > 24 are indicative of non-increased risk of fall, 20–24 indicate moderate risk, and <20 indicate high risk (or indicate non-ambulatory subjects if <2). It is divided into two sections: “balance” and “gait”. In “balance” section, scoring from 0 to 16, it is tested the balance in different activities, namely sitting, arising, immediate standing, sustained standing, nudging, turning 360°, sitting down; in “gait” section”, scoring from 0 to 12 it is tested the ability and the confidence in moving, examining the characteristics of the steps, the path, and the trunk. The sum of such sections gives the total score.
- CIRS [41], for the assessment of the comorbidity burden. It evaluates 14 categories of pathologies concerning some organs and systems: hypertension, cardiological, vascular, hematopoietic, respiratory, eye–ear–nose–throat–larynx, upper and lower gastroenterological, liver–pancreatic, renal, genitourinary, musculoskeletal, neurological, endocrinological, psychiatric, and behavioral diseases. Each category is given a score from 1 (minimum impairment) to 5 (severe disease or risk of life), the sum of which defines the total score. Such score can be divided for the number of categories to obtain the complex comorbidity index. The number of categories scoring 3 or more defines the severity index.
- Mini Mental State Examination (MMSE) [42], for cognitive assessment. It examines different domains (temporal orientation—from 0 to 5 points, spatial orientation—from 0 to 5 points, immediate memory—from 0 to 3 points, attention—from 0 to 5 points, delayed memory—from 0 to 3 points, language—from 0 to 7 points, and praxis—from 0 to 2 points). Conversion tables are available to avoid age or school influences. Scores < 26 are suggestive of mild-to-severe cognitive impairment [43]
- Geriatric Depression Scale (GDS) [44], for mood assessment. It is made up of 15 yes/no questions regarding satisfaction, dropped interests, happiness, boredom, good spirit, fears, subjective utility, energy, and hope. Each question is given 1 (depressed) or 0 (non-depressed) points. Scores > 5 are suggestive of deflected mood.
- Activities of Daily Living (ADL), expressed as Barthel Index, and Instrumental Activities of Daily Living (IADL) [45], for the assessment of residual autonomies. ADL evaluate feeding (from 0 to 10 points), bathing (from 0 to 5 points), grooming (from 0 to 5 points), dressing (from 0 to 10 points), urinary continence (from 0 to 10 points), bowel continence (from 0 to 10 points), toilet use (from 0 to 10 points), transfers (from 0 to 15 points), mobility (from 0 to 15), walking stairs (from 0 to 10): the sum of each defines the Barthel Index, which is higher when the level of independence in such activities is high. IADL evaluate the ability to use the telephone, shopping, food preparation, housekeeping, laundry, transportation, responsibility for medications, handling finances: each item is given 0 (dependent) or 1 point (independent).
- Mini Nutritional Assessment (MNA) [46,47], for the assessment of nutritional status. It evaluates anthropometric measures (body mass index, mid-arm, and calf circumferences), food intake, weight loss, mobility, recent stress, neuropsychological status, independence, drugs taken, pressure ulcers, as well as how may full meals are eaten daily, markers for protein, fruit, vegetables, and fluids intake, mode of feeding, and subjective view about nutritional and general status. Scores < 17 indicate malnutrition and 17–23.5 indicate risk of malnutrition.
- Number of different drugs taken.

### 2.6. Statistical Analysis

Variables were expressed as medians and interquartile ranges (IQR) or in percentages (%), where appropriate. The Kolmogorov–Smirnov test was used to test normal distribution. Spearman’s coefficient of rank correlation (rho) was used to correlate the PPT and POMA scores. The Kruskal–Wallis test for independent sample was used to compare the groups on dependent variables. The Conover test was used for post-hoc analysis. Kaplan–Meier curves were designed in order to estimate the survival probability: their results were expressed as hazard ratios (HRs), for the comparison of which the Log-rank test was used. The multivariate analysis was conducted with a logistic regression—stepwise (*p*-values > 0.1 excluded by the model), the results of which were expressed as odds ratios (ORs), and Area Under the Receiver Operating Characteristic (ROC) curve (AUC).

The results are reported indicating *p*-values in reference to 95% confidence interval (CI).

MedCalc software (Version 20.218, Ostend, Belgium) was used for the statistical analysis.

## 3. Results

The study included 384 community-dwelling people aged 65 years or more, of whom 280 were women (72.7%), with a median age of 81 years. The characteristics of the sample are shown in Table 1 and Table 2.

**Figure 1:**
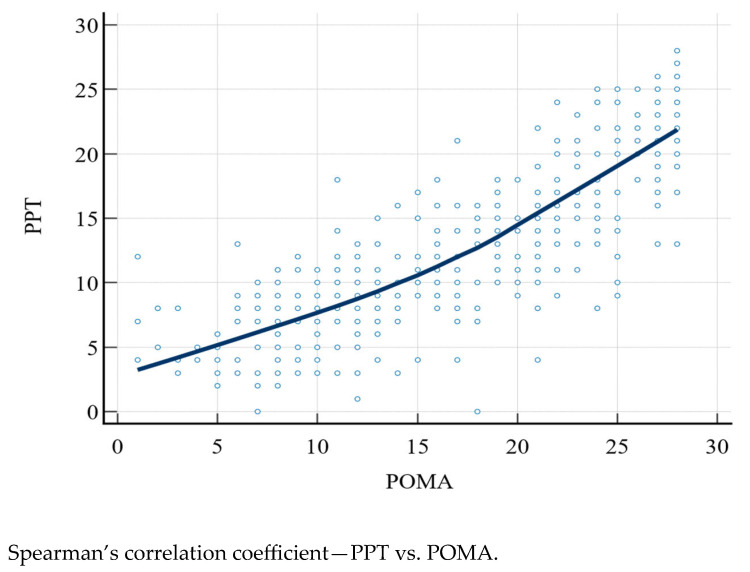
PPT, Physical Performance Test; POMA, Performance Oriented Mobility Assessment.

The PPT and POMA scores were compared with Spearman’s correlation coefficient (rho), which was 0.828 (95% CI: 0.793–0.857, *p* < 0.0001) (Figure 1).

We divided the sample into three groups, according to the abovementioned scores, thus obtaining group 1 (PPT ≥ 20 and POMA > 24, 43 subjects), group 2 (PPT of 11–19 and/or POMA of 20–24, 92 subjects), and group 3 (PPT < 11 and/or POMA < 20, 249 subjects). As in Table 3, the age was the only variable with no significant difference in the groups (*p* = 0.913). ADL, IADL, and MNA scores were significantly lower in group 3 than in group 2, and also in group 2 than in group 1 (*p* < 0.0001); similarly, the GDS scores were significantly higher in group 3 than in group 2, and in group 2 than in group 1 (*p* < 0.0001). The CIRS scores (*p* < 0.0001) and number of drugs taken (*p* = 0.0009) were significantly higher in group 3 than in the other two groups. Finally, the MMSE scores were higher in group 1 than in the other two groups (*p* = 0.001).

**Figure 2:**
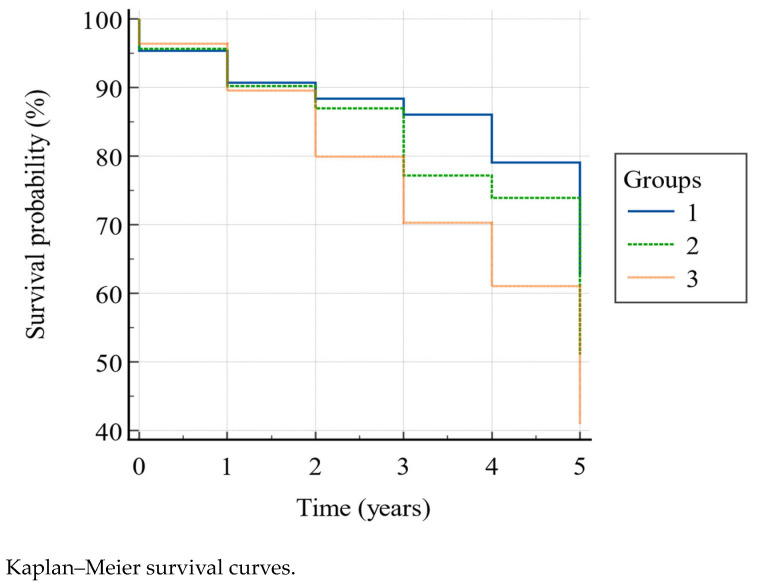
Group 1, PPT ≥ 20 and POMA > 24; group 2, PPT between 11–19 and/or POMA between 20–24; group 3, PPT < 11 and/or POMA < 20.

According to the Kaplan–Meier model (Figure 2) the overall 5-year mortality was 54.2%, and the survival rate was significantly higher (Log-rank χ^2^ = 8.78, *p* = 0.0124) in group 1 than in group 2 and 3, and in group 2 than in group 3, as shown in Table 4, in the second (88.4% vs. 87% vs. 79.9%), third (86% vs. 77.2% vs. 70.3%), fourth (79.1% vs. 73.9% vs. 61%), and fifth (62.8% vs. 51.1% vs. 41%) year. In particular, belonging to group 1 gave HR = 1.83 (95% CI: 1.20–2.79) for survival with respect to group 3. The other HRs showed a higher tendency to survive in group 1 vs. group 2, and in group 2 vs. group 3, though without reaching the statistical significance (Table 5).

These data were deepened with a multivariate analysis, in which we considered death as a dependent variable, and age, CIRS, MMSE, GDS, ADL, IADL, MNA, and number of drugs taken as independent variables. With an AUC = 0.723 (standard error: 0.028, 95% CI: 0.67–0.77, *p* < 0.0001), the logistic regression considered age (OR: 1.08), GDS (OR: 0.89), and MNA (OR: 0.84) independently associated with the outcome. The other variables were excluded by the model (Table 6).

## 4. Discussion

Global aging is a pressing issue and is expected to lead to an increasing number of social and health implications [1,8]. Poor physical performance and high risk of falling are associated with negative outcomes [19,20,21,22], especially in older people. Indeed, even if such risks and outcomes can also depend on cognitive-affective status, comorbidity burden, and polypharmacotherapy [24], several studies focus on the assessment of physical performance and risk of falling [25,26,27], based on balance, gait, and the execution of standardized physical tasks.

The primary aim of our study was to identify if physical performance and risk of falling are related to mortality. Its secondary aim was to establish if people with both severely impaired physical performance, and high risk of falling also presented impairment in other geriatric domains. We recruited 384 subjects aged 65 years or more, of whom 280 were women (72.7%), with a median age of 81 years, and followed them for five years. We decided to exclude people with CIRS > 30, meaning a significant comorbidity burden, in order to avoid potential confounding factors: it was in fact demonstrated that comorbidity burden is an independent risk factor for deaths [49]. We did not exclude any particular pathology, although there are several studies demonstrating the association between specific comorbidities and death, but they are usually mediated by a general burden, typical of the elderly age [50,51,52,53].

Our data showed a strong correlation between physical performance and risk of falling (rho = 0.828), emphasizing that in older subjects, various domains follow the same trend, and a person with the inability to carry out standardized activities also presents a higher risk of falling. Accordingly, we divided the sample into three groups: the first, made up of people without an augmented risk of falling and able to perform adequate physical activity, the second, made up of people with a moderate risk of falling and/or disability, and the third, made up of people with a severe risk of falling and/or disability. Our analysis suggested that people belonging to the third group, in addition to the worse physical abilities, also revealed higher comorbidities (though our sampling foresaw too high of a comorbidity burden, in order to avoid confounding factors), worse cognitive capacities, more deflected mood, reduced autonomies, and more deficient nutritional status, according to the above. Moreover, the prospective study showed that poor physical capacities and a high risk of falling were associated with an increased risk of death, with the survival curves spreading with increasing follow-up months, reaching 41% 5-year survival in severely impaired people, 51.1% survival in moderately impaired people, and 62.8% survival in people with adequate physical abilities and a falling risk. In order to deepen such results, we conducted a multivariate analysis to consider a possible relationship between death and impaired geriatric domains, highlighting age, mood, and nutritional status as significant regressors of the outcome. In particular, older age showed the lowest hazard ratio, while poorer nutritional status (116%) and better mood (111%) were more clearly independently associated with death. If the direct proportionality with age and worse nutritional status is consistent with the literature [54,55], the association with mood is not [56]. We believe that, although significative, it could not be associated with an effective clinical difference between the patients, owing to the fact that the GDS median score was moderately high (8 points), and the larger part of the sample (59.1%) had deflected mood, especially the most physically impaired subjects.

The strengths of our study are represented by the 5-year follow-up, and the fact that it was performed using common and easy-to-administer screening tools, also easily reproducible in everyday clinical practice. It did not take into account some variables, such as fear of falling [57], sarcopenia [58], and lack of exercise [59], which were indeed reported to influence the results, and this is its main limitation. Future prospective studies are needed to confirm our results, and public health intervention is necessary to reduce the risk of falling among old subjects.

## 5. Conclusions

In conclusion, our study tries to address the necessity of screening for risk of falling [60,61,62], continuing along the path traced by other studies, demonstrating that poor physical performance combined with a high risk of falling, correlated with each other, are associated with a higher mortality and impairment in multiple domains in older adults.

## Figures and Tables

**Table 1:** Characteristics of the sample (multidimensional assessment).

Variable	Minimum	Maximum	Median	IQR	Kolmogorov-Smirnov
Age (years)	65	97	81	76–86	**0.0113**
PPT	0	28	11	8–17	**<0.0001**
POMA	1	28	17	11–23	**<0.0001**
CIRS	18	30	28	26–29	**<0.0001**
MMSE	0	30	21.9	16.9–25.5	**0.0001**
GDS	0	15	8	4–11	**<0.0001**
ADL	5	100	79.5	61–90	**<0.0001**
IADL	0	8	2	1–4	**<0.0001**
MNA	8	30	21	18–24	**<0.0001**
Drugs taken (n.)	0	18	5	4–8	**<0.0001**

IQR, interquartile range; PPT, Physical Performance Test; POMA, Performance Oriented Mobility Assessment; CIRS, Cumulative Illness Rating Scale; MMSE, Mini Mental State Examination; GDS, Geriatric Depression Scale; ADL, Activities of Daily Living; IADL, Instrumental Activities of Daily Living; MNA, Mini Nutritional Assessment; n., number.

**Table 2:** Prevalence of comorbidities.

Comorbidities	Percentage
Hypertension	63.9%
Previous myocardial infarction	5.2%
Peripheral vascular disease	23.4%
Chronic cerebrovascular disease	23.2%
Atrial fibrillation	8.6%
Anemia *	25.3%
Chronic obstructive pulmonary disease	14.1%
Osteoarthritis	56.5%
Osteoporosis	38%
Chronic kidney disease	6.5%
Diabetes mellitus	17.4%

* Hb < 13 g/dL [48].

**Table 3:** Comparison between groups.

Variable	Group 1 (n. 43)		Group 2 (n. 92)		Group 3 (n. 249)		Kruskal–Wallis	Conover
	Median	IQR	Median	IQR	Median	IQR	*p*-Value	Different from
Age (years)	80	76–85	80	75–86	81	77–85	0.913	-
								-
								-
CIRS	26	23–28	26	24–28	28	27–30	**<0.0001**	1 from 3
								2 from 3
								3 from 1 and 2
MMSE	25.4	20.7–28.3	20.7	16.4–25.4	21	16.5–25	**0.001**	1 from 2 and 3
								2 from 1
								3 from 1
GDS	3	1–6	6	3–10	9	6–11	**<0.0001**	1 from 2 and 3
								2 from 1 and 3
								3 from 1 and 2
ADL	98	92–100	89	83–96	67	50–81	**<0.0001**	1 from 2 and 3
								2 from 1 and 3
								3 from 1 and 2
IADL	6	5–7	3	1–5	2	1–3	**<0.0001**	1 from 2 and 3
								2 from 1 and 3
								3 from 1 and 2
MNA	25	22–27	23	20–25.5	20.5	17–22.5	**<0.0001**	1 from 2 and 3
								2 from 1 and 3
								3 from 1 and 2
Drugs taken (n.)								1 from 3
	4	3–7	5	2–7	6	4–8	**0.0009**	2 from 3
								3 from 1 and 2

Group 1, PPT ≥ 20 and POMA > 24; group 2, PPT of 11–19 and/or POMA of 20–24; group 3, PPT < 11 and/or POMA < 20; IQR, interquartile range; PPT, Physical Performance Test; POMA, Performance Oriented Mobility Assessment; CIRS, Cumulative Illness Rating Scale; MMSE, Mini Mental State Examination; GDS, Geriatric Depression Scale; ADL, Activities of Daily Living; IADL, Instrumental Activities of Daily Living; MNA, Mini Nutritional Assessment; n., number.

**Table 4:** Survival rates.

Survival Time (Years)	Group 1		Group 2		Group 3		Overall	
	Survival Proportion	Standard Error	Survival Proportion	Standard Error	Survival Proportion	Standard Error	Survival Proportion	Standard Error
0	0.953	0.032	0.957	0.021	0.964	0.012	0.961	0.009
1	0.907	0.044	0.902	0.031	0.896	0.019	0.898	0.015
2	0.884	0.049	0.870	0.035	0.799	0.025	0.826	0.019
3	0.86	0.053	0.772	0.044	0.703	0.029	0.737	0.022
4	0.791	0.062	0.739	0.046	0.610	0.031	0.661	0.024
5	0.628	0.074	0.511	0.052	0.410	0.031	0.458	0.025

Group 1, PPT ≥ 20 and POMA > 24; group 2, PPT of 11–19 and/or POMA of 20–24; group 3, PPT < 11 and/or POMA < 20.

**Table 5:** Hazard ratios with 95% CI (survival).

	Group 1	Group 2	Group 3
Group 1	-	1.37	**1.83**
		0.86–2.20	**1.20–2.79**
Group 2	0.72	-	1.3328
	0.45–1.16		0.97–1.84
Group 3	**0.54**	0.75	-
	**0.36–0.83**	0.54–1.03	

Group 1, PPT ≥ 20 and POMA > 24; group 2, PPT of 11–19 and/or POMA of 20–24; group 3, PPT < 11 and/or POMA < 20.

**Table 6:** Logistic regression—stepwise (dependent variable: exitus).

Variable *	OR	95% CI	*p*-Value
Age (years)	1.08	1.04–1.12	**0.0001**
GDS	0.89	0.83–0.95	**0.0006**
MNA	0.84	0.79–0.90	**<0.0001**

* CIRS, MMSE, ADL, IADL, drugs taken (n.) excluded from the model (*p* > 0.1). OR, odds ratio; CI, confidence interval; CIRS, Cumulative Illness Rating Scale; MMSE, Mini Mental State Examination; GDS, Geriatric Depression Scale; ADL, Activities of Daily Living; IADL, Instrumental Activities of Daily Living; MNA, Mini Nutritional Assessment; n., number.

## Data Availability

The data and materials used and/or analyzed during the current study are not publicly available. The are available from the corresponding author upon reasonable request.

## Abbreviations

ADL, Activities of Daily Living; CIRS, Cumulative Illness Rating Scale; GDS, Geriatric Depression Scale; IADL, Instrumental Activities of Daily Living; MMSE, Mini Mental State Examination; MNA, Mini Nutritional Assessment; POMA, Performance Oriented Mobility Assessment; PPT, Physical Performance Test
